# Structural insights of two novel N-acetyl-glucosaminidase enzymes through in silico methods

**DOI:** 10.3906/kim-2006-19

**Published:** 2020-12-16

**Authors:** Arif Sercan ŞAHUTOĞLU, Hatice DUMAN, Steven Alex FRESE, Sercan KARAV

**Affiliations:** 1 Department of Chemistry, Faculty of Arts and Sciences, Çanakkale Onsekiz Mart University, Çanakkale Turkey; 2 Department of Molecular Biology and Genetics, Faculty of Arts and Sciences, Çanakkale Onsekiz Mart University, Çanakkale Turkey; 3 Evolve Biosystems, Inc., Davis, CA USA; 4 Department of Food Science and Technology, University of Nebraska, Lincoln, NE USA

**Keywords:** *N* -glycan, endo- *β* - *N* -acetylglucosaminidase, EndoBI-1, EndoBI-2

## Abstract

EndoBI-1 and EndoBI-2 are two endo- *β-N-* acetylglucosaminidase isoenzymes that cleave *N-N’-* diacetylchitobiosyl moieties found in various types of native *N* -glycans. These *N* -glycans are indigestible by human infants and adults due to the lack of responsible glycosyl hydrolases and they act as selective prebiotics for a probiotic microorganism, *Bifidobacterium longum* subsp *. infantis* , in the large intestine. The selectivity and the thermostability of EndoBI-1 and EndoBI-2 suggest that these enzymes may be useful for many scientific and industrial applications. In this study, the growing numbers of homologous sequences in different databases were exploited in a comparative approach to investigate structural properties of EndoBI-1 and EndoBI-2 enzymes. Moreover, the complete and partial homology models of these two enzymes were generated and evaluated. Selected models were used for docking studies of the plus subsite ligand of these enzymes for further understanding on the substrate selectivity of EndoBI enzymes.

## 1. Introduction

Mannosyl-glycoprotein endo- *β* - *N* -acetylglucosaminidases or simply endo- *β* - *N* -acetylglucosaminidases (ENGase, EC 3.2.1.96) are glycoside hydrolyses which hydrolyze the *N* , *N* ′-diacetylchitobiosyl moieties in glyco-peptides/proteins containing the -[Man(GlcNAc)2]Asn- unit in their core structure [1]. Upon endo-hydrolysis with ENGase enzymes, one *N* -acetyl-D-glucosamine (GlcNAc) remains attached to the glyco-peptide/protein and the remaining native oligosaccharide is released. Therefore, ENGase enzymes are considered as one of the best ways to obtain native glycans from glyco-peptides/proteins and considered as invaluable tools of glycan technology [2].

**Figure 1:**
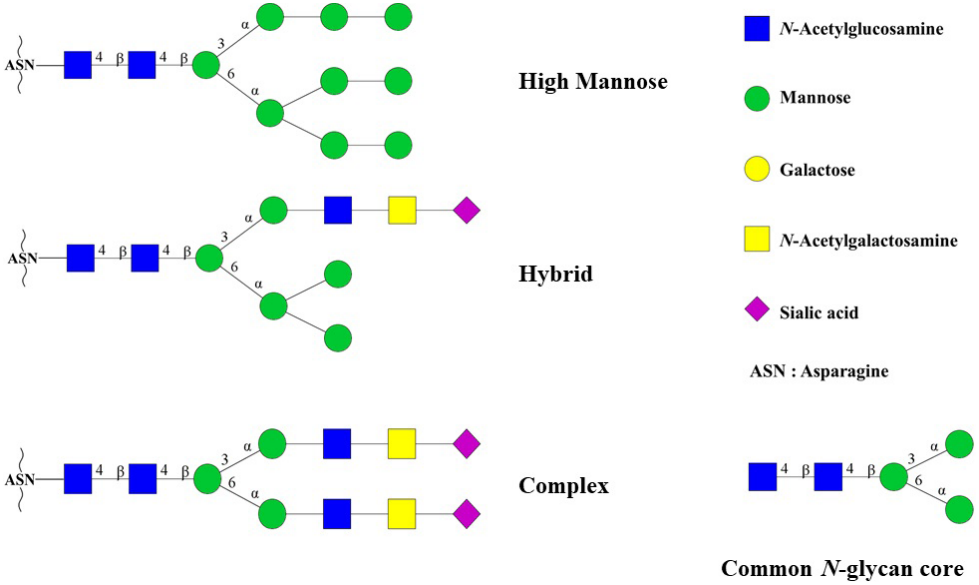
Representation of common N-glycan cores and different types of N-glycan structures. The figure was prepared with GlycoWorkbench software (v2.1, release 146).

ENGases (EndoBI-1 and EndoBI-2) of *Bifidobacterium longum* subsp *. infantis* ( *B. infantis* ) are novel enzymes which are capable of cleaving *N* ‐ *N* ′‐diacetylchitobiosyl moieties of the core of high mannose (HM), complex type (CT), and hybrid (HY) *N* ‐glycans (Figure 1). These *N* ‐glycan molecules have been shown to act as novel prebiotics that stimulate the growth of probiotic bacteria *B. infantis* [3]. Moreover, EndoBI-1 and EndoBI-2 may have different distribution between infants and adults, which may further suggest the importance of these enzymes in healthy gut microbiome formation in both adults and infants.

EndoBI-1 and EndoBI-2 and are unique amongst other ENGase members. The enzymes are produced by an anaerobic gram-positive bacterium, *B. infantis* , which is a natural member of healthy infant gut microbiome [4]. Therefore, both enzymes are considered as safe for use in food and pharmaceutical industries even for infant products. Considering that the source of similar ENGase enzymes are mostly pathogens that use these enzymes for host immune system evasion, these two enzymes are the only safe alternatives for such applications yet. Moreover, thanks to being prokaryotic members of the GH18 family, these enzymes could be easily cloned and/or mass-produced with known microbiologic procedures and industrial techniques. EndoBI-1 and EndoBI-2 cleave *N* ‐glycans without perturbating the native glycan structure [5]. The enzymes are considered as fucose (Fuc)-tolerant, meaning their activity is not affected by the fucosylation of the *N* ‐glycan core and therefore have a wider substrate specificity than similar enzymes [6]. Both enzymes are active towards all major types of *N* -linked glycans found in glycol-peptides/proteins. Last but not least, both enzymes are heat-resistant up to 95 °C [7], in contrast to other commercially available ENGases such as PNGase F.

Although the structures of various ENGases have been studied [8], currently there is no structural information on EndoBI-1 and EndoBI-2 enzymes in Protein Data Bank (PDB). However, bioinformatic investigation together with comparative modeling could provide invaluable structural information on these two enzymes. The obtained structural information could be used to explain the unique properties of these enzymes. Proper structure/function relationship information could further be used to discover other enzymes with similar properties and unique specificities from either healthy human microbiome or other sources such as bee microbiome. Moreover, GH18 members are known to be modular enzymes which have high potential in protein engineering studies [9]. Therefore, structure information on these enzymes could be used to generate modified ENGase enzymes that could be used in various fields as versatile glycobiology tools.

In this study, the complete and partial comparative models of EndoBI-1 and EndoBI-2 enzymes were generated, compared, and evaluated. Selected models were further used for docking studies of the plus subsite ligand, Fuc-GlcNAc, for further understanding on the wide substrate selectivity and the structural basis for the Fuc tolerance of EndoBI enzymes.

## 2. Materials and methods

### 2.1. Database search and sequence alignment

EndoBI-1 and EndoBI-2 target sequence homologs in different databases were searched using BLAST [10]. The multiple sequence alignments were performed with Clustal-Omega software which was run on The European Molecular Biology Laboratory *(* EMBL *)* web server [11]. Easy Sequencing in PostScript (ESPript-3.0) [12] program was used to render sequence similarities and secondary structure information from aligned sequences. Sequence logos of the alignments were prepared with WebLogo 3 web-based application [13].

### 2.2. Comparative modelling

The crystal structures of Endo-COM (PDB: 6KPL) from *Cordyceps militaris* was selected as the template [8]. The complete homology models of the enzymes were generated using the ROBETTA web server u207f [14,15]. The glycosidase domains of the enzymes were modelled with I-TASSER web server https://zhanglab.ccmb.med.umich.edu/I-TASSER/ [16]. The reliabilities of the templates and models were verified by SAVES web server http://servicesn.mbi.ucla.edu/SAVES/ of UCLA-DOE-LAB using PROCHECK [17], WHATCHECK [18], ERRAT [19], VERIFY 3D [20,21], and PROVE [22] programs. Structural Alignment of Multiple Proteins (STAMP) program of MultiSeq module of VMD http://www.ks.uiuc.edu/Research/vmd/ was used to superpose the structures [23]. Structures of templates and models were analyzed and summarized with PDBsum database [24].

### 2.3. Ligand docking

Docking of ligand was performed with SwissDock http://www.swissdock.ch/ [25] molecular docking server based on docking software EADock DSS [26]. *2* -acetamido- *2* -deoxy- *6* - *O* -(α- *L* -fucopyranosyl)- *D* -glucopyranose [Fuc-GlcNAc] was used as Fuc containing “plus” subsite ligand [8]. Ligand preparation including drawing, displaying, and characterizing structures and substructures, energy minimization, adding polar hydrogens and assigning Gasteiger charges to all atoms of the ligand were performed with Marvin 17.6, 2017, ChemAxon http://www.chemaxon.com. Docking preparation for protein models and evaluation of dock results were performed with UCSF-Chimera http://www.rbvi.ucsf.edu/chimera software [27].

### 2.4. Molecular dynamic simulations

All molecular dynamics (MD) simulations were performed using the Nanoscale Molecular Dynamics (NAMD) software [28] using CHARMM 36 [29] all-atom force field [30] including the correction maps [31]. The solvation and the ionization of the enzyme partial models and docked complexes were performed using VMD. The water molecules were described by the TIP3P model [32]. The isothermal-isobaric (NPT) simulations were performed at 1.0 atm pressure and 310 K temperature with 2-fs time steps using Langevin pressure coupling. Periodic boundary conditions were applied along x, y, and z directions during the simulations. Electrostatic interactions were calculated by using the particle-mesh Ewald (PME) method [33]. The visualization and analysis of MD trajectories were performed with VMD. The models and the ligand complexes were subjected to equilibration and energy minimization through (10 ns) MD simulations with regular (2 ps) minimization intervals via greedy algorithm. All simulations were repeated in triplicate using different velocity seeds and the average results were reported for all cases.

## 3. Results

### 3.1. The overall 3D structure

**Figure 2:**
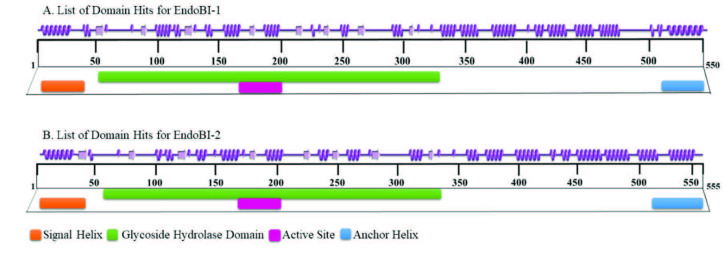
Graphical summaries of secondary structures (PDBsum) together with conserved domain information (CDD) of the EndoBI-1 and EndoBI-2 homology models.

EndoBI-1 enzyme includes a signal helix (1-36), the active ENGase (37-517), and a transmembrane helix (518-545). Similarly, EndoBI-2 enzyme includes a signal peptide (1-60), the active ENGase (61-515), and a gram-positive LPXTG cell wall anchor helix (520-555) (Figure 2).

The masses of the complete enzymes were calculated as 56.1 and 59.6 kDa, respectively, with Compute pI/Mw tool of SIB ExPASy Bioinformatics Resource Portal.

The ENGase parts of the enzymes consist of two discrete domains similar to other GH18 members. First, there is an *N* -terminal glycoside hydrolase (GH) domain for both enzymes. The GH domain of EndoBI-1 resides between amino acid residues 51–366 whereas GH domain of EndoBI-2 resides between amino acid residues 61–360. The second domain of the enzymes is a substrate-binding domain which contains a potential carbohydrate binding module (CBM) and a 4-helix up-down bundle motif which is similar to other members of the GH18 family. Due to the lack of high homology templates for this domain, the complete models could not be generated with SWISSMODEL or I-TASSER servers. Therefore, the complete models of the enzymes were generated through a hybrid modelling approach. The *N* -terminal signal and *C* -terminal anchor helixes were deleted and the enzymes were remodelled using ROBETTA web server. The domains were parsed with Ginzu protocol and GH domain was modelled with homology modelling whereas CBM domain was modelled with ab initio modelling.

The validations of the full models were performed with SAVES server. According to VERIFY results, the 95.3% and 98.0% of the amino acid residues have averaged 3D-1D score ≥0.2 for EndoBI-1 and EndoBI-2 enzymes, respectively. The overall quality factors were calculated with ERRAT and found to be 90.2% and 95.4%, respectively.

The Ramachandran maps were created by PROCHECK. The Ramachandran plots show ≥95% of the amino acids in the most favored regions and only ≤0.6 in the disallowed regions, which suggests reasonably good overall structures for both enzymes.

**Figure 3:**
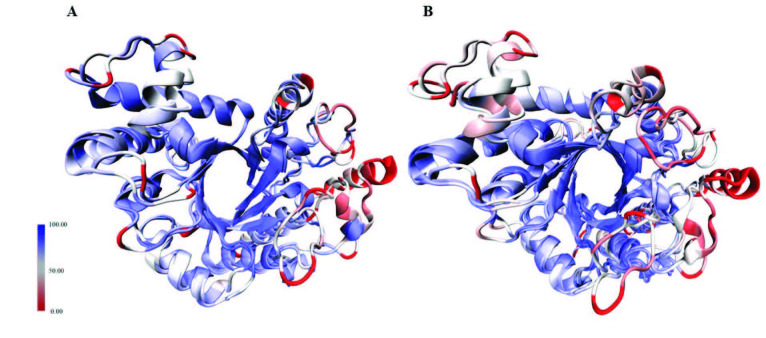
Structural alignments of the GH domains of A) EndoBI-1 and EndoBI-2; and B) EndoBI-1, EndoBI-2, and the template (Endo-COM).

The structural similarities of the models and the template structures were calculated with DALI Structure Comparison Server and visualized with MultiSeq plugin of VMD. The RMSD values between the glycosidase domains of EndoBI-1 and EndoBI-2 were 1.6 Å (Figure 3A) whereas it was 8.9 Å when the glycosidase domain of their template structure (Endo-COM) was included (Figure 3B). The RMSD per residue was never more than 3.5 for EndoBI-1 and EndoBI-2 (Figure 3).

Although sequence similarities were higher with *Cordyceps militaris* chitinase (Table), Endo-COM, both for EndoBI-1 and EndoBI-2, structural alignment results were found to be higher with *Streptococcus pyogenes* ENGases EndoS and EndoS2. Structurally EndoBI-1 is found to be the most similar to the EndoS (PDB ID: 4NUY) [34] (Dali Z-Score = 30.2, RMSD = 3.5, %ID = 25) whereas EndoBI-2 is found to be the most similar to the EndoS2 [35] Dali Z-Score = 33.7, RMSD = 2.2, %ID = 24).

**Table:** Table. Alignment results for EndoBI-1 (A) and EndoBI-2 (B) sequences in PDB database.

Query account verifier	Subject account verifier	% identity	E value	Bit score	% positives
ACJ53522.1	6KPL_A	32.414	1.05x10-30	122	47.93
	6KPN_A	31.724	9.26x10-30	119	47.93
	6E58_A	27.986	5.64x10-16	82.4	44.03
	4NUY_A	26.557	4.15x10-12	70.1	42.62
	4NUZ_A	26.230	2.42x10-11	67.4	42.3
	6EN3_A	25.902	6.48x10-11	66.2	42.3
	1EDT_A	25.397	1.4	32.3	40.21
	1C90_A	24.868	2.9	31.2	40.21
	1C8X_A	25.000	3.8	30.8	40.43
	1C91_A	24.868	3.9	30.8	40.21
	1C3F_A	24.868	4.0	30.8	40.21
	1C92_A	24.868	4.4	30.8	39.68
	4AXN_A	27.907	9.9	29.6	45.35

Query account verifier	Subject account verifier	% identity	E value	Bit score	% positives
BAJ71450.1	6KPL_A	34.114	1.90x10-36	138	49.16
	6KPN_A	33.445	2.04x10-35	135	49.16
	6E58_A	25.949	4.06x10-15	79.7	44.62
	4NUY_A	24.262	2.94x10-10	64.3	42.95
	4NUZ_A	23.934	1.49x10-9	62.0	42.62
	6EN3_A	23.607	5.13x10-9	60.5	42.62
	3IAN_A	25.581	0.88	33.1	43.02
	4W5Z_A	33.663	3.7	31.2	47.52
	4HMC_A	33.663	6.1	30.8	47.52

### 3.2. The glycoside hydrolyse domain

The GH domain models of the EndoBI enzymes were refined via MD simulations. RMSD, RMSF, and Rg patterns of the models over MD trajectories were given in the supplementary information (Figure S1). EndoBl-1 and EndoBI-2 glycosidase domains adopt the (β/α)8 u2016 (TIM) barrel conformation like other typical bacterial ENGases. Structurally, EndoBI-1 glycosidase domain is the most similar to the analogous domain of EndoS (Dali Z-Score = 41.1, RMSD = 1.6, %ID = 24; whereas EndoBI-2 glycosidase domain to the analogous domain of Endo-COM (Dali Z-Score = 39.6, RMSD = 0.9, %ID = 32). Although the TIM barrel structure is quite similar, loops that link the structure which is known to modulate the selectivity of the enzymes has considerable versatility among ENGase enzymes [36]. The loops surrounding the active site forms a Y-shaped structure which interacts with the “plus” and “minus subsites of the substrate. Perpendicular part of the Y-shape interacts with the “plus” subsite of the substrate which is the common glycan core whereas the fork part interacts with the “minus” subsite of the substrate which is an HM, HY, or CT leaving group. The active sites of the enzymes are located close to the center of the barrel structure.

### 3.3. Carbohydrate binding module domain

The second domain of EndoBI-1 and EndoBI-2 contains a unique helix bundle that contains 4 up-down alpha helixes and a possible CBM. Due to the lack of high homology template structures in PDB, these domains were modelled with rather high RMSD in full models generated with ROBETTA relative to the GH domains which has quite low RMSD values and modelled with both ROBETTA and I-TASSER. The domain extends away from the glycosidase domain and in this manner resembles the *C* -terminal domains of EndoS and EndoS2. Similarly, being not packed extensively against glycosidase domain and exhibiting 2388 Å2 and 3009 Å2 (for EndoBI-1 and EndoBI-2 respectively calculated with CASTp) of buried surface (pocket Richards’ solvent accessible) area suggest that these domains could potentially rotate to translate in a more compact conformation when the substrate is bound just like *C* -terminal domains of EndoS and EndoS2. Similar to the EndoS and EndoS2, EndoBI-1 and EndoBI-2 CBMs may have a considerable effect on substrate selectivity [34], and facilitate enzymatic activity [35]. Likewise, EndoBI-1 and EndoBI-2 GH domains have possibly evolved to work their corresponding CBMs [35]. Domain swapping studies should be performed on EndoBI-1 and EndoBI-2 for further understanding of this similarity.

According to CAZy database ENGases with the EC number of 3.2.1.96 contain members of CBM families 32 and 50. Although similar ENGases such as EndoS and EndoS2 have not been formally assigned to a CBM family yet, they are proposed to contain CBM 32 scaffolds [35]. CBM 32 is described as a diverse domain family frequently found in bacterial enzymes that interact with human *N* -glycans. CBM 32 family preferentially recognizes the nonreducing terminus of GlcNAc [37] and usually have a bound structural calcium ion [38].

Family 32 usually binds *N* -glycans with the help of the solvent exposed aromatic amino acid residues on the loops of the *β* -sandwich scaffold [39]. Although complete models of the enzymes generated in this study show high overall quality, both models have failed to represent *β* -sandwich scaffold of the CBM 32 domains. EndoBI-2 is currently under the CBM 32 family entry according to CAZy but there is no information for EndoBI-1 yet. However, it would be safe to propose the existence of a CBM 32 for both enzymes mainly due to the activity of the enzymes on human *N* -glycans just like EndoS and EndoS2. X-ray diffraction studies of the EndoBI-1 and EndoBI-2 enzymes or high quality templates for these enzymes are required for further evaluation of CBMs of these enzymes.

### 3.4. Possible reaction mechanism

**Figure 4:**
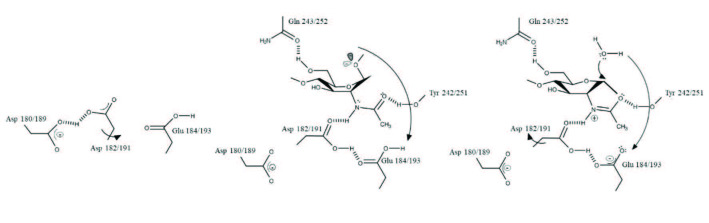
Proposed mechanism for EndoBI-1 and EndoBI-2 enzymes. Amino acid residues are numbered for EndoBI-1 and EndoBI-2 respectively.

The majority of the GH18 members in the Protein Data Bank (PDB) are chitinases (EC 3.2.1.14), which endolytically cleave *β* -1,4 glycosidic bonds of the chitin releasing oligomeric, dimeric (di-GlcNAc), or monomeric (GlcNAc) products or *N* -acetylhexosaminidases (EC 3.2.1.52) which could further cleave di-GlcNAc to GlcNAc [9]. However, ENGases (3.2.1.96) such as EndoBI-1 and EndoBI-2 only cleave *N* , *N* ′-diacetylchitobiosyl unit in common *N* -glycan core of glycoproteins. The key catalytic residues of EndoBI-1 and EndoBI-2 enzymes are found in a conserved (LIVMFY)-(DN)-G-(LIMFY)-(DN)-(LIMFY)-DN)-X-E motif similar to other GH18 Enzymes [9]. The catalytic residues of EndoBI-1 and EndoBI-2 are identical as **L-D-G-L-D-I-D-M-E** , which is a highly conserved motif among other ENGases with known protein structure. This similarity suggests a closer evolutionary relationship for ENGases of GH18 family. The glutamic acid residues (E184 and E193 for EndoBI-1 and EndoBI-2, respectively) of the motif may act as general acid proton donors. These Glu residues may protonate the glycosidic bond oxygen to assist the departure of the leaving group, aglycone. Then these residues may act as general bases to deprotonate the nucleophilic water during the oxazolinium ion intermediate hydrolysis (Figure 4) [40]. Aspartic acid (D182 and D191 for EndoBI-1 and EndoBI-2, respectively) is found as an additional catalytic carboxylate in D-M-E motif [41]. The physical separation of the catalytic residues D and E results with the E not being able to act as a nucleophile without assistance. Therefore, the assistance of the neighbor amino acid residues (D182 and D191) may be required to deprotonate the *N* -acetamido nitrogen of the substrate nucleophile during oxazolinium ion formation/breakdown of the double displacement [42].

The single point mutation of the catalytic Glu residue of the EndoBI-1 (E184→N) results in complete activity loss. E184 shows significant binding affinity to the *α* -6-fucosylated *N* -glycan core, which is characteristic for human *N* -linked glycoproteins [4]. The active site equivalent of this amino acid in EndoBI-2 is E193. This general acid/base catalytic amino acid may be replaced with glutamine (Q) in some plants members of the GH18 family. These proteins lack enzyme activity and function as enzyme inhibitors or lectins [43]. Such single point mutations may be used to produce catalytically inactive ENdoBI-1 and EndoBI-2 mutants for affinity purification purposes in glycobiology.

### 3.5. The docking studies

**Figure 5:**
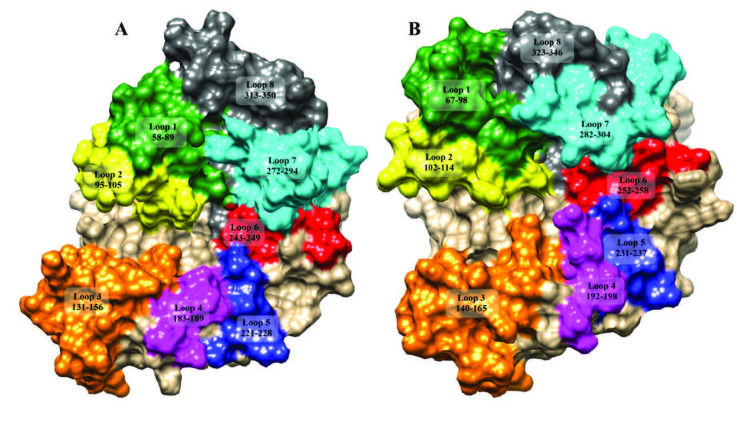
Loop organization of EndoBI-1 (A) and EndoBI-2 (B) enzymes.

The basic 3D structure of the TIM barrel and the position of the catalytic residues are similar in both EndoBI-1 and EndoBI-2 and conserved among other ENGases. However, the loops connecting the barrel helixes differ considerably from other enzymes. The loop displaying system [8,35,36] was used to illustrate the structural differences of EndoBI-1 and EndoBI-2 (Figure 5).

**Figure 6:**
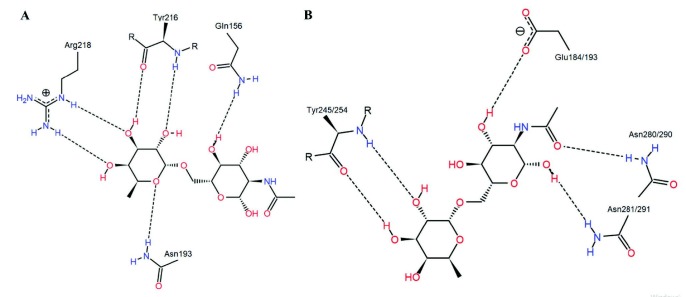
PoseView images of ligand bound template mutant (D154N/E156Q) crystal structure (6KPN) (A) and EndoBI dock result (B).

**Figure 7:**
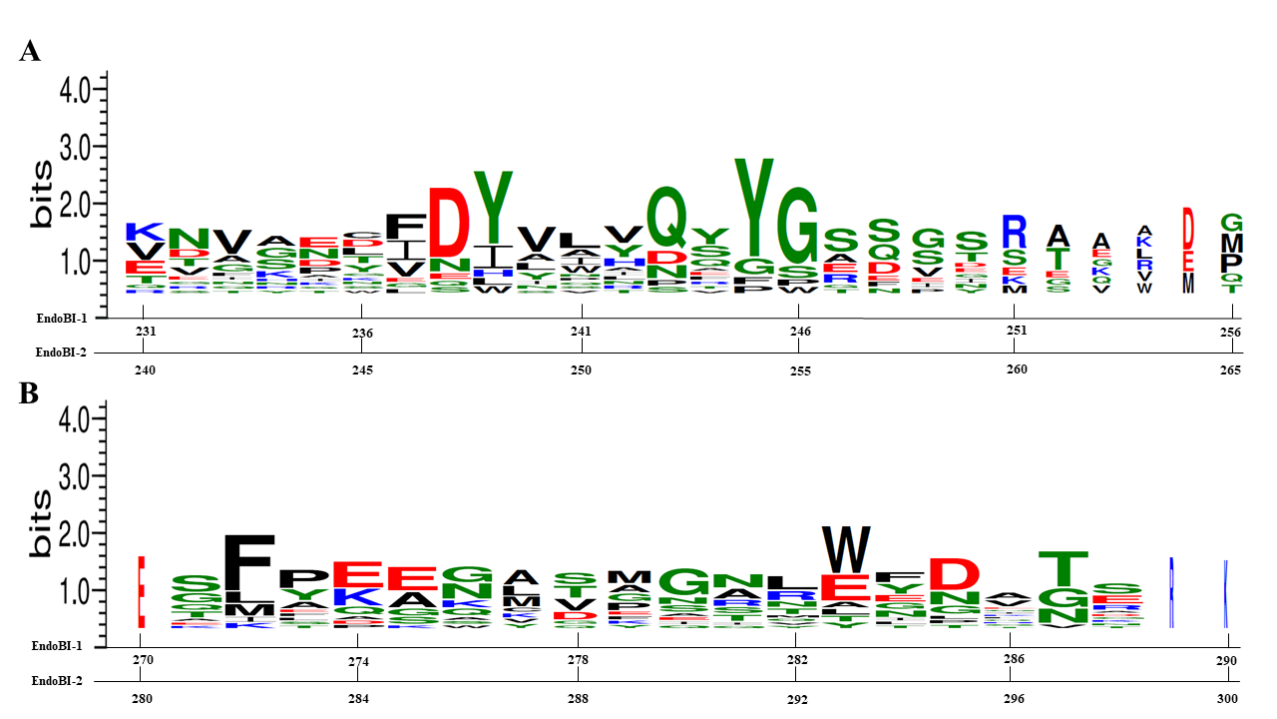
Sequence logos of the amino acid residues responsible for the +1’ (A) and +1 (B) subsites’ substrate recognition for ENGase enzymes.

According to the loop displaying system, plus subsites of the EndoBI-1 and EndoBI-2 are located in loop 6 whereas minus subsite is located near loop 1 and loop 2. Fuc-GlcNAc was docked to the plus subsites of the EndoBI-1 and EndoBI-2 GH domain models to investigate the recognition for the protein side part of the *N* -glycan substrates. Docking studies were performed by using OE atoms of E184 and E193, respectively, as center in a 10 Å3 grid box and with 3 Å flexibility. Endo-COM docking studies were performed as positive control and the results were compared with the crystal structure (6KPN) [8]. After the docking studies, the clusters with the highest full-fitness values were selected and MD simulations were performed to assess the GH domain model–ligand stability. RMSD, RMSF, and Rg patterns of the complexes and RMSD patterns of the ligands were provided in the supplementary information (Figures S2 and S3, respectively). The average atomic distance between the ASP182/191 OD atoms of the models and the LIGH21 atoms of the ligands over MD trajectories were also provided in the supplementary information (Figure S4). The dock results of Endo-COM were coherent with the crystal structure information (6KPN) (Figure 6). Fuc resides in +1’ side cleft in loop 6 whereas GlnNAc resides +1 side cleft and the substrate interacts with the enzyme via H bonds. The Fuc moiety forms multiple H bonds with the R groups of the N193 and Y216 together with the backbone of R218. The GlcNAc moiety forms multiple H bonds with the R groups of the Q156 and possibly W243. Instead of the Q214-S215-Y216-G217-R218 motif of the Endo-COM, EndoBI enzymes have the conserved Q(243/252)-Q-Y(245/254)-G84(246/255)-S motif which leads 2 fewer H bonds at +1’ recognition site. On the other hand, the R groups of the Asn dimer of the conserved E-E-G-D-M-N(280/290)-N(281-291)-R-W motif form H bonds with GlcNAc which leads two additional H bonds at +1 recognition site. This motif is not conserved in the template structure. The subtle changes in +1 and +1’ recognition sites may determine the affinity differences between multiple ENGase enzymes. The modification of these sequences (Figure 7) may directly affect the substrate binding affinity of the EndoBI enzymes which may have great effects in molecular genetic studies.

## 4. Discussion

EndoBI-1 and EndoBI-2 enzymes are unique thermostable ENGases with broad selectivity. Thanks to these properties they have great potential as glycobiology tools. Moreover, these enzymes are produced by a food-safe microorganism, which allows great possibilities in the food industry, especially in the infant product market. Although the biochemical properties of these enzymes have been studied, the lack of structural information hampers further enzyme engineering studies.

EndoBI enzymes with different substrate-binding affinities may be obtained with different strategies in further studies. The modular nature of EndoBI enzymes could be exploited to exchange the CBMs of these enzymes with other known CBMs of GH18 family. The plus subsite carbohydrate binding motifs of these enzymes could be modified to increase the substrate binding affinity of the enzymes. Moreover, the modification of the active site of the EndoBI enzymes may be performed to generate catalytically inactive proteins that could be used as selective glycan binding tools for purification purposes.

Although this work provides valuable insights for the structure/function relationship and the plus subsite substrate recognition of EndoBI enzymes, high quality crystal structures are still needed especially for the minus subsite recognition and the thermostability studies. The leaving parts of the bi/tri-antennary glycan substrates recognized at the minus subsite are usually too big for proper docking studies. Therefore, the minus subsite recognition studies need high quality X-ray structures with minus subsite ligands. The CBM motifs usually associated with substrate and calcium ion binding and therefore considered to have great effects on the selectivity and the thermostability of ENGase enzymes. Although complete models of the enzymes generated in this study show high overall quality, both models have failed to represent *β* -sandwich scaffold of the CBM 32 domains due to lack of suitable template structures. Therefore, CBMs of EndoBI enzymes could be considered as unique among other structurally known ENGases. The structure of this domain may be effective on the unique thermostability of these enzymes. Therefore, the comparison of X-ray structures with/without docked calcium ions and complete glycan structures may provide unique information on the reasons that lay beside the thermostability of these enzymes.

Supplementary Materials

## References

[ref1] (2009). The X-ray crystal structure of an Arthrobacter protophormiae Endo-β-N-acetylglucosaminidase reveals a (β/α)8 catalytic domain, two ancillary domains and active site residues key for transglycosylation activity. Journal of Molecular Biology.

[ref2] (2017). The ENGases: Versatile biocatalysts for the production of homogeneous: N-linked glycopeptides and glycoproteins. Chemical Society Reviews.

[ref3] (2015). Characterizing the release of bioactive N-glycans from dairy products by a novel endo-β-N-acetylglucosaminidase. Biotechnology Progress.

[ref4] (2012). Endo-β-N-acetylglucosaminidases from infant gut-associated bifidobacteria release complex N-glycans from human milk glycoproteins. Molecular & Cellular Proteomics.

[ref5] (2015). Kinetic characterization of a novel endo-beta-N-acetylglucosaminidase on concentrated bovine colostrum whey to release bioactive glycans. Enzyme and Microbial Technology.

[ref6] (2015). A novel endo‐β‐N‐acetylglucosaminidase releases specific N‐glycans depending on different reaction conditions. Biotechnology Progress.

[ref7] (2014). Bacterial glycosidases in pathogenesis and glycoengineering. Future Microbiology.

[ref8] (2019). Structural basis for the specific cleavage of core-fucosylated N-glycans by endo-N-acetylglucosaminidase from the fungus Cordyceps militaris. Journal of Biological Chemistry.

[ref9] (2009). Evolution of family 18 glycoside hydrolases: Diversity, domain structures and phylogenetic relationships. Journal of Molecular Microbiology and Biotechnology.

[ref10] (1990). Basic local alignment search tool. Journal of Molecular Biology.

[ref11] (2013). Analysis tool web services from the EMBL-EBI. Nucleic Acids Research.

[ref12] (2014). Deciphering key features in protein structures with the new ENDscript server. Nucleic Acids Research.

[ref13] (2004). WebLogo: A sequence logo generator. Genome Research.

[ref14] (2004). Protein structure prediction and analysis using the Robetta server. Nucleic Acids Research.

[ref15] (2004). Protein structure prediction using Rosetta. Methods in Enzymology.

[ref16] (2010). A unified platform for automated protein structure and function prediction. Nature Protocols.

[ref17] (1993). PROCHECK: A program to check the stereochemical quality of protein structures. Journal of Applied Crystallography.

[ref18] (1996). Errors in protein structures. Nature.

[ref19] (1993). Verification of protein structures: Patterns of nonbonded atomic interactions. Protein Science.

[ref20] (1992). Assessment of protein models with three-dimensional profiles. Nature.

[ref21] (1991). A method to identify protein sequences that fold into a known three-dimensional stucture. Science.

[ref22] (1996). Deviations from standard atomic volumes as a quality measure for protein crystal structures. Journal of Molecular Biology.

[ref23] (1996). Visual molecular dynamics. Journal of Molecular Graphics.

[ref24] (2001). PDBsum: summaries and analyses of PDB structures. Nucleic Acids Research.

[ref25] (2011). a protein-small molecule docking web service based on EADock DSS. Nucleic Acids Research.

[ref26] (2011). Fast docking using the CHARMM force field with EADock DSS. Journal of Computational Chemistry.

[ref27] (2004). UCSF Chimera - A visualization system for exploratory research and analysis. Journal of Computational Chemistry.

[ref28] (2005). Scalable molecular dynamics with NAMD. Journal of Computational Chemistry.

[ref29] (2009). CHARMM: The biomolecular simulation program. Journal of Computational Chemistry.

[ref30] (1998). All-atom empirical potential for molecular modeling and dynamics studies of proteins. The Journal of Physical Chemistry B.

[ref31] (2004). Improved treatment of the protein backbone in empirical force fields. Journal of the American Chemical Society.

[ref32] (2004). A modified TIP3P water potential for simulation with Ewald summation. The Journal of Chemical Physics.

[ref33] (1995). A smooth particle mesh Ewald method. The Journal of Chemical Physics.

[ref34] (2014). Crystal structure of Streptococcus pyogenes EndoS, an immunomodulatory endoglycosidase specific for human IgG antibodies. Proceedings of the National Academy of Sciences.

[ref35] (2019). Molecular basis of Broad spectrum N-glycan specificity and processing of therapeutic IgG monoclonal antibodies by Endoglycosidase S2. American Chemical Society Central Science.

[ref36] (2018). Structural basis for the recognition of complex-type N-glycans by Endoglycosidase S. Nature Communications.

[ref37] (2009). Portrait of an enzyme, a complete structural analysis of a multimodular β-N-acetylglucosaminidase from Clostridium perfringens. Journal of Biological Chemistry.

[ref38] (2011). Structural analysis of a putative family 32 carbohydrate-binding module from the Streptococcus pneumoniae enzyme EndoD. Acta Crystallographica Section F: Structural Biology and Crystallization Communications.

[ref39] (2008). Insight into ligand diversity and novel biological roles for family 32 carbohydrate-binding modules. Molecular Biology and Evolution.

[ref40] (1996). The pK(a) of the general acid/base carboxyl group of a glycosidase cycles during catalysis: A 13C-NMR study of Bacillus circulans xylanase. Biochemistry.

[ref41] (2004). Mutational and computational analysis of the role of conserved residues in the active site of a family 18 chitinase. European Journal of Biochemistry.

[ref42] (2010). Visualizing the reaction coordinate of an O-GlcNAc hydrolase. Journal of the American Chemical Society.

[ref43] (1995). Crystal structure of concanavalin B at 1.65 Å resolution. Journal of Molecular Biology.

