# Supplementary material for: Structural insights of two novel N-acetyl-glucosaminidase enzymes through in silico methods
Source: Turk J Chem. 2020 Dec 16;44(6):1703–12. doi: 10.3906/kim-2006-19 (PMC7763110; doi:10.3906/kim-2006-19)
Supplement: Supplementary file 1 — Supplementary Materials [file turkjchem-44-1703-sup001.pdf]

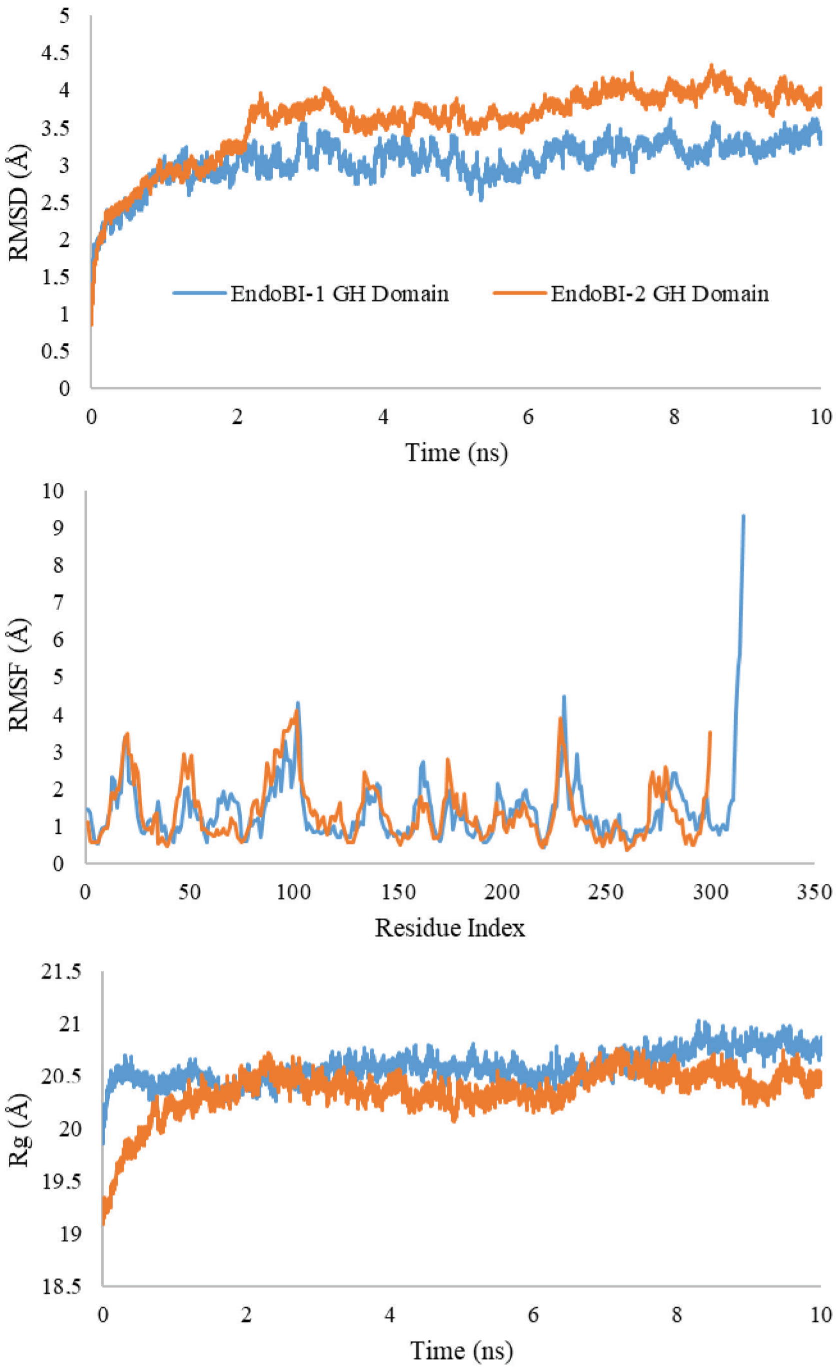

Figure S1. RMSD, RMSF, and Rg patterns of the EndoBI GH domain models over MD trajectories during refinement.

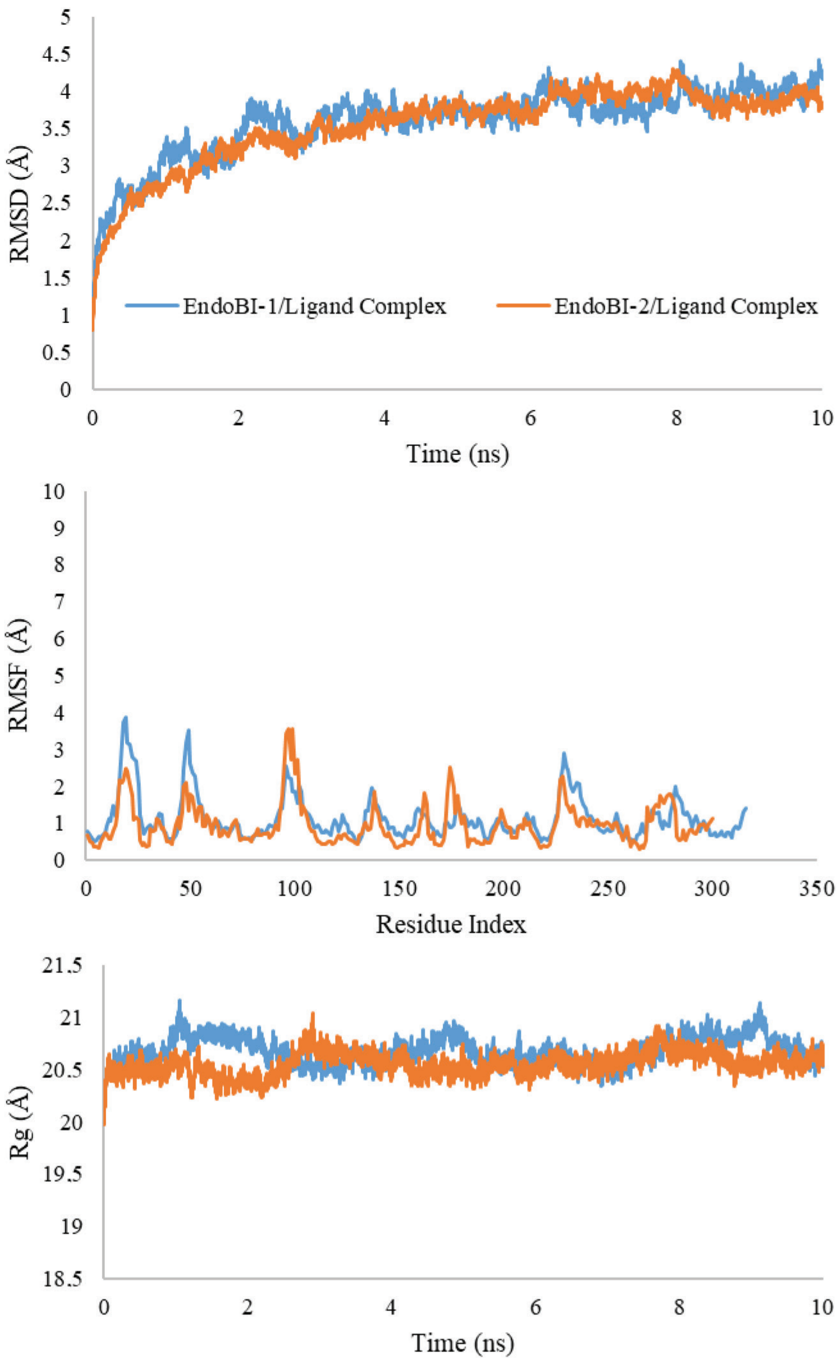

Figure S2. RMSD, RMSF and Rg patterns of the EndoBI GH domain – ligand complexes over MD trajectories.

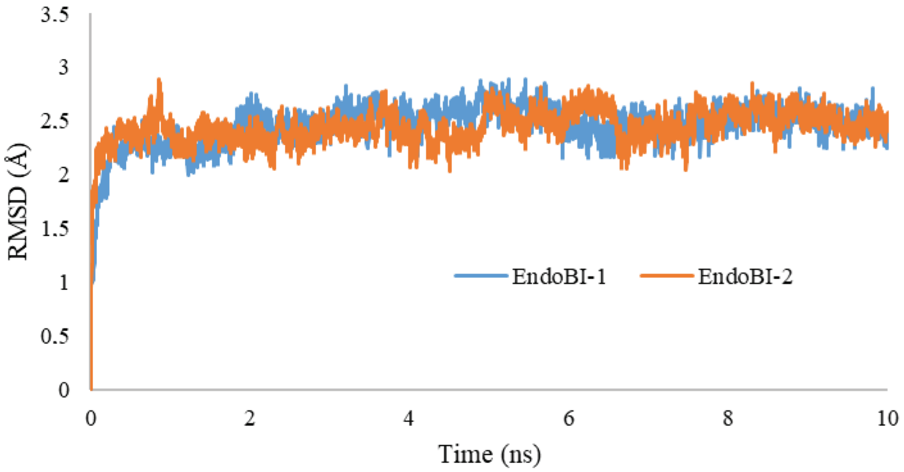

Figure S3. RMSD patterns of the bound ligands over MD trajectories.

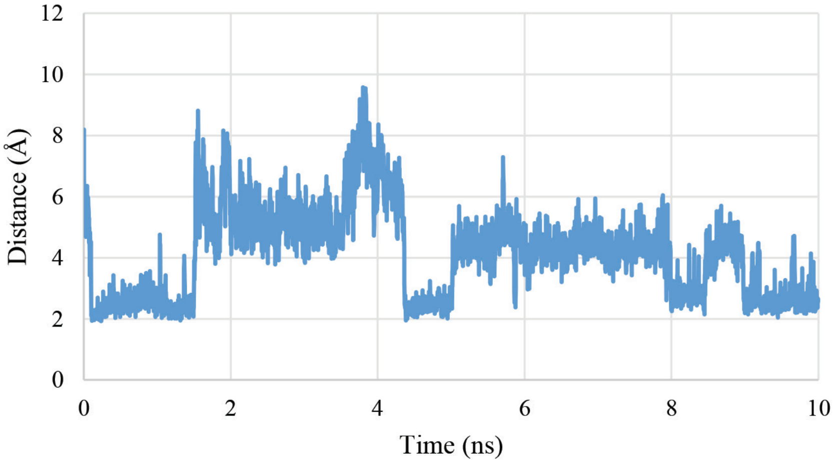

Figure S4. Average distance between ASP182/191 OD atoms and LIGH21 atoms over MD trajectories.
